# Prevalence and risk factors of preoperative venous thromboembolism in patients with malignant musculoskeletal tumors: an analysis based on D-dimer screening and imaging

**DOI:** 10.1186/s12959-022-00382-2

**Published:** 2022-04-26

**Authors:** Kenta Hayashida, Yusuke Kawabata, Keiju Saito, Shintaro Fujita, Hyonmin Choe, Ikuma Kato, Masanobu Takeyama, Yutaka Inaba

**Affiliations:** 1 Department of Orthopaedic Surgery, Yokohama City University, 3-9 Fukuura, Kanazawa-ku, Yokohama, 236-0004 Japan; 2 Department of Molecular Pathology, Yokohama City University, Yokohama, Japan

**Keywords:** Preoperative venous thromboembolism, VTE, Deep vein thrombosis, DVT, Malignant musculoskeletal tumor, Malignant musculoskeletal tumor, D-dimer

## Abstract

**Background:**

Venous thromboembolism (VTE) is a major complication in patients with malignant tumors and orthopedic disorders. Although it is known that patients undergoing surgery for malignant musculoskeletal tumor are at an increased risk of thromboembolic events, only few studies have investigated this risk in detail. Therefore, the aim of this study was to determine the prevalence and risk factors for preoperative VTE in malignant musculoskeletal tumors patients.

**Methods:**

We retrospectively reviewed the medical records of 270 patients who underwent surgical procedures, including biopsy for malignant musculoskeletal tumor, have undergone measurements of preoperative D-dimer levels, and were subsequently screened for VTE by lower extremity venous ultrasonography and/or contrast-enhanced computed tomography scans. Statistical analyses were performed to examine the prevalence and risk factors for VTE. Receiver operating characteristic (ROC) analysis was performed to verify the D-dimer cutoff value for the diagnosis of VTE.

**Results:**

Overall, 199 patients (103 with primary soft tissue sarcomas, 38 with primary bone sarcomas, 46 with metastatic tumors, and 12 with hematologic malignancies) were included. D-dimer levels were high in 79 patients; VTE was detected in 19 patients (9.5%). Multivariate analysis indicated that age ≥ 60 years (*P* = 0.021) and tumor location in the lower limbs (*P* = 0.048) were independent risk factors for VTE. ROC analysis showed that the D-dimer cutoff value for the diagnosis of VTE was 1.53 µg/mL; the sensitivity and specificity were 89.5% and 79.4%, respectively.

**Conclusions:**

Our study indicated that age and tumor location in the lower limbs were independent risk factors for preoperative VTE in malignant musculoskeletal tumors patients. D-dimer levels were not associated with VTE in the multivariate analysis, likely because they are affected by a wide variety of conditions, such as malignancy and aging. D-dimer is useful for exclusion diagnosis because of its high sensitivity, but patients with high age and tumor location in the lower limbs are a high-risk group and should be considered for imaging evaluation such as ultrasonography regardless of D-dimer levels.

**Trial registration:**

Our study was approved by the institutional review board. The registration number is B200600056. The registration date was July 13, 2020.

**Supplementary Information:**

The online version contains supplementary material available at 10.1186/s12959-022-00382-2.

## Background

Venous thromboembolism (VTE), including deep vein thrombosis (DVT) and pulmonary embolism (PE), is a major complication in orthopedic surgery. VTE is also known to be associated with malignancy, which increases the risk of VTE by 2- to sevenfold [1–3]. Several mechanisms may be involved in the promotion of thromboembolic events in cancer patients [4]. Because the risk of VTE is reported to be particularly high during the first few months after the diagnosis of malignancy [3], it is important to assess affected patients regarding this condition before initiating treatment.

D-dimer, the breakdown product of stabilized fibrin, is frequently used to screen for VTE as there is well-established evidence that levels below 0.5 µg/mL have an exceedingly high negative predictive value for the exclusion of PE [5–7]. Although lower extremity venography is the mainstay for the diagnosis of DVT, it has recently been replaced by lower extremity venous ultrasonography and contrast-enhanced computed tomography (CT) [8–10]. The combination of clinical symptoms, D-dimer concentrations, and contrast-enhanced CT has made it possible to diagnose 97.9% of VTE cases [11, 12].

The prevalence of perioperative VTE in malignant musculoskeletal tumors patients ranges from 2.7% to 22% [13–21]. A myriad of factors, such as age, size and location of the tumor, chemotherapy, Ewing sarcoma family of tumors, metastases, pathological fracture, and type of surgery, have been suggested to be related to the occurrence of VTE [13–17, 20–23]. However, most previous studies analyzed postoperative, rather than preoperative, patients who underwent orthopedic surgery [13, 15–18, 22]. In addition, routine screening for VTE was not considered; therefore, the reported rate of VTE represented only those that were clinically symptomatic [15, 17].

Because such limitations may cause an underestimation of the prevalence of VTE, only limited evidence is available for understanding preoperative VTE and associated risks for malignant musculoskeletal tumors patients. Therefore, we investigated the prevalence and risk factors for VTE in these patients and hypothesized that a combination of measuring D-dimer levels and imaging-based examination may be the best screening strategy for VTE.

## Methods

**Fig. 1:**
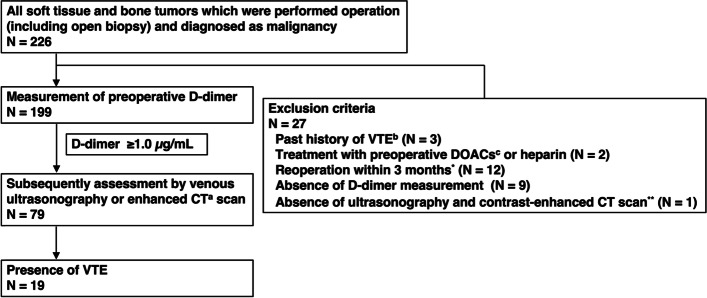
Flow chart depicting the venous thromboembolism assessment strategy. Patients were evaluated using D-dimer screening, lower extremity venous ultrasonography, and contrast-enhanced computed tomography. A total of 23 patients were not included based on the exclusion criteria. *Additional extensive resections for unplanned resections or positive margins and non-orthopedic surgery patients were included. **Only cases with D-dimer > 1.0 μg/ml and not yet evaluated imaging studies were indicated. **a** CT: computed tomography; **b** VTE: venous thromboembolism; **c** DOACs: direct oral anticoagulant

After obtaining approval from our institutional review board, we retrospectively reviewed the medical records of patients treated at our institution between January 2014 and June 2020. Out of 270 malignant musculoskeletal tumors patients who underwent orthopedic surgical procedures, including open biopsy, and were assessed for preoperative VTE according to a flowchart developed by our institution, a total of 199 patients were included (Fig. 1). VTE was screened by measuring preoperative D-dimer levels and subsequently performing lower extremity venous ultrasonography and/or contrast-enhanced CT. D-dimer levels were measured by LPIA D-dimer (Mitsubishi Chemical Medience, Tokyo, Japan); owing to the sensitivity of this assay, levels < 0.50 µg/mL were considered 0.50 µg/mL. Ultrasonography and CT scans were performed if the D-dimer level was ≥ 1.0 µg/mL. In this study, DVT in the lower extremities that involved the popliteal vein or those above was defined as the proximal type; DVT involving the area below the popliteal vein was defined as the distal type.

The clinical profiles of patients with VTE were retrospectively assessed. The anatomical location of the tumor was defined as the lower limb, including the pelvis, hip, thigh, knee, lower leg, ankle, and foot. Tumor location was also assessed based on the depth of the tumor. A soft tissue tumor located on the surface of the fascia was defined as superficial. Soft tissue tumors deeper than the fascia and bone tumors were defined as deep.

The Mann–Whitney test was used for continuous variables, and the chi-squared test or Fisher’s exact test were used for categorical variables. Logistic regression analysis was used for the multivariate analysis. Receiver operating characteristic (ROC) analysis was performed to verify the cutoff value of D-dimer for the diagnosis of VTE. Statistical significance was set at *P* < 0.05. All statistical analyses were performed using R (R Foundation for Statistical Computing, Vienna, Austria).

## Results

We excluded patients if they met any of the following criteria: benign condition; history of VTE; treatment with direct oral anticoagulants or heparin before the assessment of VTE; reoperation within 3 months; absence of preoperative D-dimer measurements; and absence of lower extremity venous ultrasonography or contrast-enhanced CT examinations in cases with D-dimer > 1.0 μg/ml. Baseline information was summarized in Table 1. The patients comprised 113 men and 86 women, with a mean age of 60.9 years (range, 14–91 years). The tumor location was the lower limb in 133 patients. The D-dimer concentration was ≥ 1.0 µg/mL in 79 (39.7%) patients. The overall prevalence of preoperative VTE detected by ultrasonography and contrast-enhanced CT scans was 9.5% (19 patients). VTE was detected in 8.7% and 7.9% of patients with soft tissue and bone sarcomas, respectively. The prevalence of VTE in patients with metastases was 10.9%. The tumors comprised 103 primary soft tissue sarcomas, 38 primary bone sarcomas, 46 metastatic tumors, and 12 hematologic malignancies. The pathological diagnoses of the patients are summarized in Table 2. Proximal VTE and PE were detected in 3 (2.5%) and 2 (1.7%) patients, respectively. Most patients had asymptomatic VTE, but 3/19 patients (15.8%) had local edema or pain. The clinical profiles of the patients with VTE are summarized in Table 3. The patients comprised 10 men and 9 women with a mean age of 59.7 years (range, 19–91 years). The tumor was located in the lower limbs in 17 patients, accounting for 89.5% of the patients with VTE. The mean D-dimer level was 2.64 µg/mL (range, 1.05–11.72 µg/mL). ﻿Among the patients with VTE, 9 had soft tissue sarcomas and 3 had bone sarcomas. The cases of soft tissue sarcoma included 4 cases of myxofibrosarcomas, 3 undifferentiated pleomorphic sarcomas (UPSs), and 2 leiomyosarcomas. The tumor was located on the surface of the fascia in 4 patients and deeper than the fascia in 5 patients. The cases of bone sarcoma included 2 UPSs of the bone and 1 osteosarcoma. Seven patients had bone metastases and hematologic malignancies. ROC analysis showed that the cutoff value of D-dimer for the diagnosis of VTE was 1.53 µg/mL using by Youden's index; the area under the curve was 0.857 (95% CI, 0.804—0.911), and the sensitivity and specificity were 90.0% and 79.6%, respectively.

**Table 1:** Demographic and clinical variables for patients

Variable		Number of patients		Univariate analysis			Multivariate analysis		
		VTE (-)	VTE ( +)	Odds ratio	95% confidence interval	*p*-value	Odds ratio	95% confidence interval	*p*-value
Number of patients (*n* = 199)		180	19 (9.5%)						
Diagnosis	Soft tissue sarcoma	94	9 (8.7%)						
	Bone sarcoma	35	3 (7.9%)						
	Metastasis	41	5 (10.9%)						
	Hematologic malignancy	10	2 (16.7%)						
Sex	Male	103	10						
	Female	77	9	0.81	0.28–2.31	0.85^a^			
Age (years)		59.7 ± 1.4	70.8 ± 3.7						
	< 60	74	2						
	≧ 60	106	17	5.89	1.33–54.1	0.011^a^	5.80	1.32–26.5	0.021^c^
BMI (cm/kg)		22.46	21.39			0.24^b^			
Size (mm)		80.4	65.8			0.79^b^			
Location	Lower limb	116	17						
	Other	64	2	4.66	1.05–42.9	0.038^a^	4.56	1.01–20.4	0.048^c^
Origin	Bone	80	10						
	Soft	100	9	0.72	0.25–2.08	0.63^a^			
	Subcutaneous ~ fascia	36	4						
	Fascia ~ bone	144	15	1.14	0.26–3.90	0.77^a^			
D-dimer		0.65	2.64			> 0.001^b^	1.01	0.96–1.08	0.51^c^
Performance Status	0–2	154	16						
	3–4	26	3	1.11	0.19–4.29	0.74^a^			
Pathological fracture		23	3	1.28	0.22–4.99	0.71^a^			
Hematologic status	Hemoglobin (g/dL)	12.9	13			0.88^b^			
	White blood cell (/μL)	7010	6080			0.38^b^			
	Platelet (× 10^4^/μL)	28	26.2			0.99^b^			
	C related protein (mg/L)	1.78	0.56			0.80^b^			

^a^Fisher’s exact test

^b^Mann-Whitney U test

^c^Logistic regression analysis

**Table 2:** Diagnosis of all patients in this study

Tumor origin		Pathological diagnosis	Number of patients
Primary	Bone	Chondrosarcoma	15
		Osteosarcoma	13
		UPS	5
		Ewing sarcoma	2
		Chordoma	2
		Malignant giant cell tumor of bone	1
		Subtotal	38
	Soft tissue	Myxofibrosarcoma	27
		UPS	24
		Myxoid liposarcoma	10
		MPNST	8
		Synovial sarcoma	7
		Dedifferentiated liposarcoma	6
		Leiomyosarcoma	7
		Extraskeltal myxoid chondrosarcoma	5
		Dermatofibrosarcoma protuberans	3
		Pleomorphic liposarcoma	2
		Rhabdomyosarcoma	2
		Others	2
		Subtotal	103
Metastasis		Renal cell cancer	16
		Lung cancer	11
		Prostatic cancer	5
		Breast cancer	3
		Esophageal cancer	2
		Colorectal cancer	2
		Bladder cancer	2
		Thyroid cancer	1
		Gastric cancer	1
		Liver cancer	1
		Pancreatic cancer	1
		Uterine cancer	1
		Subtotal	46
Hematologic malignancy		Lymphoma	9
		Multiple myeloma	3
		Subtotal	12
Total			199

*UPS* Undifferentiated pleomorphic sarcoma, *MPNST* Malignant peripheral nerve sheath tumor

**Table 3:** Characteristic of patients with venous thromboembolism

Age		Sex	BMI (cm/kg)	Location	Depth	Diagnosis	Pathological fracture	PS	D-Dimer (μg/mL)	Location of VTE
1	76	Male	22.3	Thigh	Superficial	Myxofibrosarcoma	-	0	1.05	Distal
2	66	Male	18.7	Femur	Bone	Lung cancer	-	2	5.69	Distal
3	66	Male	29.1	Thigh	Superficial	UPS	-	0	1.58	Distal
4	86	Female	22.2	Thigh	Deep	Leiomyosarcoma	-	1	3.69	Proximal
5	79	Male	29.2	Lower leg	Superficial	UPS	-	1	2.16	Distal
6	77	Female	17.0	Patella	Bone	Malignant lymphoma	-	2	2.33	PE, distal
7	58	Male	19.8	Femur	Bone	Lung cancer	+	3	11.72	PE, distal
8	73	Female	16.4	Femur	Bone	UPS	+	4	2.43	Distal
9	64	Female	18.0	Tibia	Bone	Uterine cancer	-	1	4.65	Distal
10	91	Male	20.4	Forearm	Superficial	Myxofibrosarcoma	-	1	2.32	Distal
11	78	Female	20.2	Pelvis	Bone	UPS	-	1	4.23	Proximal
12	67	Female	17.9	Buttocks	Deep	UPS	-	1	2.71	Distal
13	76	Female	25.0	Lower leg	Deep	Leiomyosarcoma	-	1	1.21	Distal
14	19	Male	22.2	Tibia	Bone	Osteosarcoma	-	1	1.53	Distal
15	91	Female	20.4	Elbow	Deep	Myxofibrosarcoma	-	1	2.49	Distal
16	60	Female	23.2	Femur	Bone	Breast cancer	+	4	9.28	Distal
17	75	Male	24.5	Pelvis	Bone	Malignant lymphoma	-	1	1.76	Distal
18	85	Male	15.4	Sacrum	Bone	Lung cancer	-	1	2.85	Distal
19	79	Male	24.8	Lower leg	Deep	Myxofibrosarcoma	-	1	1.91	Proximal

*BMI* Body mass index, *PS* Performance status, *VTE* Venous thromboembolism, *UPS* Undifferentiated pleomorphic sarcoma

﻿The risk factors for VTE were analyzed using univariate analysis; we found that age ≥ 60 years (*P* = 0.011), tumor location in the lower limbs (*P* = 0.038), and D-dimer levels (*P* < 0.001) were associated with the prevalence of VTE (Table 1). In contrast, sex, body mass index, size, origin, performance status, pathological fracture, and hematologic status, including hemoglobin, white blood cell, platelet, and C-reactive protein levels, were not identified as significant risk factors. Multivariate analysis indicated that age ≥ 60 years (*P* = 0.021), and tumor location in the lower limbs (*P* = 0.048) were independent risk factors for VTE. The D-dimer level was not significant in the multivariate analysis (*P* = 0.51).

## Discussion

We investigated the prevalence of preoperative VTE in malignant musculoskeletal tumors patients. The most important finding in this study was that more affected patients could potentially have VTE than that reported by previous studies. In this study, VTE was detected in 19 of the 199 included patients (9.5%); the prevalence was therefore higher than the rate of perioperative VTE reported in previous studies [13–18]. Several conditions in our study that differed from those in previous studies may have influenced the results. First, routine screening for VTE was performed. We performed DVT assessment in combination with routine D-dimer measurement, lower extremity ultrasonography, and contrast-enhanced CT scans. This screening strategy allowed for the diagnosis of patients with asymptomatic VTE. Symptomatic VTE in this study was 3/199 patients (1.5%). The necessity of anticoagulation therapy for these patients is controversial; however, Galanaud et al. reported that patients with cancer-related isolated distal DVT had a prognosis similar to that of patients with cancer-related isolated proximal DVT and a dramatically poorer prognosis than those with isolated distal DVT without cancer [24]. In addition, Gary et al. reported Asymptomatic deep vein thrombosis and superficial vein thrombosis in ambulatory cancer patients are associated with poor survival despite anticoagulation therapy [25]. In osteoarthritis, previous report suggested that performing MDCT before and after total knee arthroplasty may be useful to clarify the incidence of VTE and to develop appropriate strategies for treatment and prevention [26]. Therefore, for the management of preoperative malignant musculoskeletal tumors patients, it is crucial to recognize that more patients have asymptomatic VTE than previously thought. Second, our study included patients with metastases and hematologic malignancies. The rate of DVT in surgical oncology patients receiving no prophylaxis was reported to be 35.2% [27]. The cancer type affects the prevalence of VTE in adolescent and young adult patients; therefore, oncology patients, except for those with sarcomas, may have a different total prevalence of VTE.

﻿Several risk factors associated with an increased susceptibility of preoperative VTE in malignant musculoskeletal tumors patients have been identified. Our study showed that increasing age and tumor location are independent risk factors. Age has been previously suggested to be associated with an increased risk of VTE [13, 20]. Kim et al. reported an odds ratio of 5.84 in patients older than 60 years [13]. Our results showed a similar trend, and only two patients with VTE were younger than 60 years of age. Patients with bone or soft tissue sarcomas located in the hip or thigh have been suggested to have an increased risk of VTE [17]. Yamaguchi et al. demonstrated a high prevalence of VTE (22%) in patients after resection of musculoskeletal tumors of the lower limb [20]. Since surgery involving the pelvis has been associated with the development of proximal DVT [22], patients with tumors in the lower limbs are suggested to be at an increased risk of VTE occurrence. In this study, 17 of 19 VTE patients had tumors in the lower limbs (89.5%), and the two VTE patients with tumors in the upper extremities were both older than 90 years of age.

Whilst the level of D-dimer showed an association with preoperative VTE in the univariate analysis, this was not confirmed in the multivariate analysis. This was likely due to the influence of increased age on D-dimer levels, as they need to be corrected by age, and the value of this fibrin degradation product itself should be evaluated carefully [5, 7]. In addition, the D-dimer cutoff value in cancer patients was reported to be higher than that commonly used in clinical practice [28]. The cutoff value of D-dimer for the diagnosis of VTE was found to be 1.53 µg/mL in the present study, which was almost the same as that used for other cancer types [29, 30]. The appropriate consideration of D-dimer limits the overuse and added cost of ultrasonography without a negative impact [31]. If D-dimer exceeds a level of 1.53 µg/mL, additional assessment of VTE by lower extremity ultrasonography and contrast-enhanced CT should be performed. In particular, patients older than 60 years and with tumor location in the lower limbs are a high-risk group and should be considered for imaging evaluation such as ultrasonography regardless of D-dimer levels.

 Our study has several limitations. First, it was retrospective in nature and had a relatively small sample size. ﻿Some predictive variables may not have had sufficient statistical power; therefore, some important variables may have been ignored. Second, patients with metastases and hematologic malignancies were included. Regarding the evaluation of VTE risk in bone and soft tissue sarcoma patients, these malignancies should be evaluated in separate categories with sufficient sample sizes. Finally, lower extremity ultrasonography and contrast-enhanced CT for VTE evaluation were not unified.

## Conclusions

Our study indicated that age and tumor location in the lower limbs were independent risk factors for preoperative VTE in malignant musculoskeletal tumors patients. D-dimer levels were not identified as independent risk factors in the multivariate analysis because it is affected by a wide variety of conditions, including malignancy and aging. D-dimer is useful for exclusion diagnosis because of its high sensitivity, but patients older than 60 years and with tumor location in the lower limbs are a high-risk group and should be considered for imaging evaluation such as ultrasonography regardless of D-dimer levels.

## Supplementary Information

**Additional file 1**.

## Data Availability

Data sharing is not applicable to this article as no datasets were generated or analysed during the current study.

## Abbreviations

CT: Computed tomography
DOAC: Direct oral anticoagulant
DVT: Deep vein thrombosis
PE: Pulmonary embolism
ROC: Receiver operating characteristic
VTE: Venous thromboembolism
