# Supplementary figures and images for: Prevalence and risk factors of preoperative venous thromboembolism in patients with malignant musculoskeletal tumors: an analysis based on D-dimer screening and imaging
Source: Thromb J. 2022 Apr 26;20:22. doi: 10.1186/s12959-022-00382-2 (PMC9040225; doi:10.1186/s12959-022-00382-2)

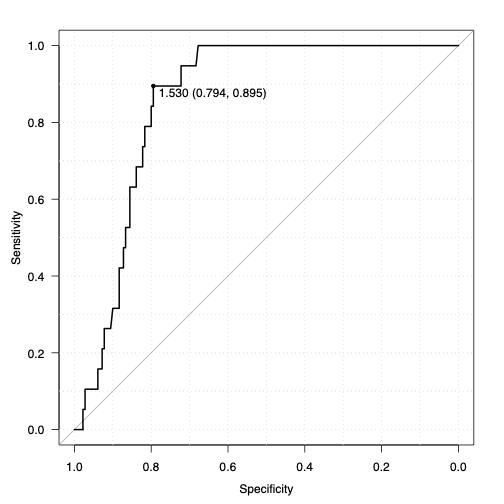

Supplement: Supplementary file 1 — Additional file 1. [file 12959_2022_382_MOESM1_ESM.tiff]
